# Analysis of the Porcine Reproductive and Respiratory Syndrome Virus Nucleocapsid Interactome

**DOI:** 10.1021/acs.jproteome.5c00121

**Published:** 2025-09-25

**Authors:** Duangnapa Kovanich, Kunjimas Ketsuwan, Kowit Hengphasatporn, Chutima Thepparit, Potchaman Sittipaisankul, Piriya Wongkongkathep, Chaitawat Sirisereewan, Navapon Techakriengkrai, Teerawut Nedumpun, Yasuteru Shigeta, Trairak Pisitkun, Sanipa Suradhat

**Affiliations:** † Center for Vaccine Development, 98841 Institute of Molecular Biosciences, Mahidol University, Nakhon Pathom 73170, Thailand; ‡ Center for Computational Sciences, 13121 University of Tsukuba, Tsukuba, Ibaraki 305-8577, Japan; § Office of Research and Innovation Affair, Institute of Molecular Biosciences, Mahidol University, Nakhon Pathom 73170, Thailand; ∥ Center of Excellence in Systems Biology, 65103 Faculty of Medicine, Chulalongkorn University, Bangkok 10330, Thailand; ⊥ Research Affairs, Faculty of Medicine, Chulalongkorn University, Bangkok 10330, Thailand; # Graduate Program in Veterinary Pathobiology, 71651 Faculty of Veterinary Science, Chulalongkorn University, Bangkok 10330, Thailand; ∇ Department of Veterinary Microbiology, Faculty of Veterinary Science, Chulalongkorn University, Bangkok 10330, Thailand; ○ Center of Excellence in Emerging Infectious Diseases in Animals, Chulalongkorn University (CU-EIDAs), Bangkok 10330, Thailand

**Keywords:** PRRSV, nucleocapsid, Arterivirus, Nidovirus, virus-host interaction

## Abstract

Porcine reproductive and respiratory syndrome virus (PRRSV) is a major swine pathogen that causes significant economic losses worldwide. The nucleocapsid (N) protein, the most abundant viral protein in infected cells, plays roles beyond its structural function, influencing various host cellular processes. Here, we report the identification of 301 cellular protein candidates interacting with PRRSV N using EGFP immunoprecipitation combined with label-free quantitative mass spectrometry. The analysis underscores the versatile nature of the N protein in targeting a wide range of cellular proteins and processes across multiple subcellular compartments. We observed strong enrichment of ribosomal proteins, nucleolar proteins involved in ribosome biogenesis, splicing factors, RNA helicases, and DNA-binding proteins involved in chromatin remodeling and DNA damage response. Additionally, we identified proteins involved in viral RNA sensing and intrinsic antiviral mechanisms that may contribute to the immunosuppressive properties of the viral protein. Several interactions were validated and further characterized for RNA dependence, including MYBBP1A, NCL, IGF2BP1, UPF3B, G3BP1, EIF2S1, RFC4, ABCF1, PPM1G, NSUN2, and NOP2. Notably, RTCB and MYBBP1A were identified as host dependency factors for PRRSV infection. Our findings expand the current understanding of PRRSV-host interactions and reveal novel N-interacting proteins that may contribute to viral pathogenesis and immune evasion.

## Graphical abstract

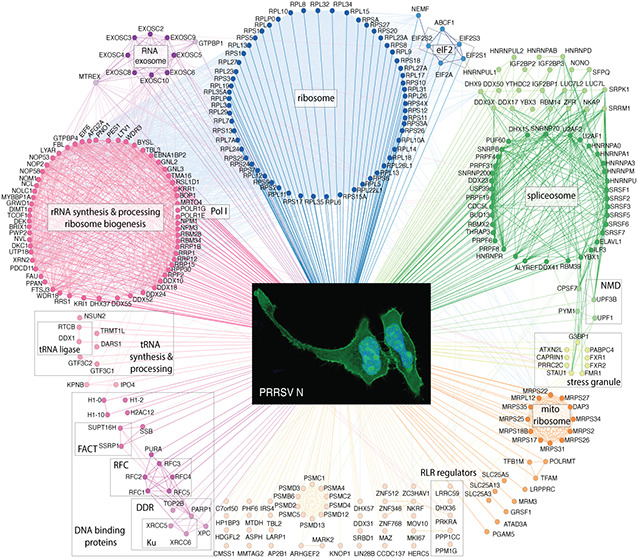

## 1 Introduction

For a successful infection, viruses have evolved various strategies to modify the host cellular environment to create a more favorable platform for their replication and assembly. These strategies typically include subversion of host immune responses, remodeling of host membranes, and manipulation of host signaling and metabolic pathways. Virus-host interactions play a critical role in mediating these processes. Indeed, viral proteins may physically interact with cellular proteins to inhibit their functions, enhance their activities, or co-opt them to support the viral replication cycle.

The porcine reproductive and respiratory syndrome virus (PRRSV) is one of the most important swine diseases, causing enormous economic losses due to reproductive failure in sows and a complex respiratory syndrome in pigs of all ages. High genetic diversity is a significant characteristic of PRRSV. Based on genomic and antigenic characteristics, PRRSV is divided into two genotypes, namely PRRSV-1 (European type) and PRRSV-2 (North American type).^1^ These two genotypes show significant genomic variation and share an overall sequence identity of only around 40–60%.^2–5^ In addition, there is significant heterogeneity among strains within a genotype, giving rise to a significant number of antigenically distinct viruses.^6^ Moreover, there are several highly pathogenic PRRSV (HP-PRRSV) strains that cause more severe reproductive and/or respiratory diseases.^7^ Another important characteristic of PRRSV is its complex interactions with the immune system, which makes the development of a vaccine against these heterologous viruses a major challenge. The virus has a very restricted tropism for only some subsets of cells of the monocytic/macrophage lineage.^8,9^ PRRSV infection elicits poor innate interferon (IFN) and cytokine responses, resulting in a weak adaptive immune response, as demonstrated by prolonged viremia and slow development of virus-specific humoral and cell-mediated immune responses (as reviewed in ref 10). The disease is now found in most pig-producing countries.

PRRSV is an enveloped single-stranded positive-sense RNA virus classified within the family *Arteriviridae*, which together with the family of coronaviruses or *Coronaviridae* and *Roniviridae* constitutes the *Nidovirales* order. The PRRSV genomic RNA (gRNA) is approximately 15 kb in size, capped on its 5′ end and polyadenylated on its 3′ terminus. The gRNA contains at least 10 open reading frames (ORFs), featuring two large overlapping open reading frames (ORF1a and ORF1b) which occupy the 5′ terminal three-quarters of the genome. The encoded polyproteins, pp1a and pp1ab, are proteolytically processed to generate at least 13–16 nonstructural proteins (NSPs) that assemble into membrane-associated replication and transcription complexes (RTCs). Viral RNA synthesis takes place within virus-induced double-membrane vesicles (DMVs) formed by extrusion of the ER membrane. The RTCs produce new gRNA and a series of subgenomic mRNA (sg mRNA) that include ORF 2–7, encoding for four membrane-associated glycoproteins (GP2a, GP3, GP4, and GP5), three unglycosylated membrane proteins (E, ORF5a, and M), and a nucleocapsid protein (N). The expression of the structural proteins is controlled at the level of sg mRNA transcription and involves the synthesis of a nested series of 3′ coterminal sg mRNA that are produced in nonequimolar amounts from the discontinuous minus-strand RNA synthesis. This mechanism of discontinuous transcription to produce a series of sg mRNA for flexible transcriptional activity is considered the transcription hallmark of the *Nidovirales* supercluster.

The 15-kDa nucleocapsid (N) protein is the product of the smallest sg mRNA (ORF7). N is the most abundant viral protein in infected cells and highly immunogenic.^11,12^ The fundamental function of the N protein is to form a viral capsid, which packages the viral genome into viral nucleocapsid that subsequently undergoes assembly with structural proteins at the ER and/or Golgi apparatus to form a new virion. The C-terminal domain is crucial for maintaining antigenic structure.^13^ The N-terminal half of the protein contains two segments enriched with basic residues, a characteristic commonly found in RNA-binding proteins.^14^ These segments are believed to facilitate binding to viral genomic RNA. In addition, the same stretches of basic residues are identified as nuclear/nucleolar localization signal (NLS/NoLS) sequence motifs.^15,16^ N dynamically shuttles between cytoplasmic and nuclear/nucleolar compartments of infected cells.^17^ The NLS-null virus-infected pigs show short duration of viremia as well as high titers of neutralizing antibodies, suggesting that the nuclear N plays a crucial role in host response and pathogenesis.^18,19^ A recent mutagenesis study has suggested an additional role of N in regulating sg mRNA transcription.^20^ In addition, N has been demonstrated to possess a regulatory function in host interferon production (as reviewed in refs 21,22 ) as well as NF-KB activation.^23^ Interestingly, the N protein possesses a unique immunomodulatory characteristic that prevents PRRSV from effective host immune clearance. N expression, both in overexpression systems and in PRRSV-infected pigs, can strongly induce anti-inflammatory cytokine interleukin-10 (IL-10) and IL-10-producing regulatory T-lymphocytes.^24^ This special immunomodulatory function contributes to the unique immunological outcome observed following PRRSV infection.

While progress has been made on the immunogenic properties of N, mainly for the purpose of vaccine development, the underlying molecular mechanisms of how N proteins modulate cellular processes to facilitate PRRSV infection and suppress antiviral innate immunity remain largely unknown. To fill this lacuna, this research utilizes a GFP-tag based affinity purification-mass spectrometry (AP-MS) approach to develop information on PRRSV N-host protein–protein interaction (PPI) network, with the aim to provide more insights into the molecular mechanisms of the infection. Here we report the identification of 301 cellular protein candidates interacting with PRRSV N proteins. The analysis underscores the versatile nature of the N protein in targeting a wide range of cellular proteins and processes in multiple subcellular compartments. We observed strong enrichment of ribosomal proteins, nucleolar proteins involved in ribosome biogenesis, splicing factors, RNA helicases, and DNA-binding proteins involved in chromatin remodeling and DNA damage response. Additionally, we identified proteins involved in viral RNA sensing and intrinsic antiviral mechanisms that may contribute to the immunosuppressive properties of the viral protein. Mining this data set, our study not only expands the current landscape of PRRSV-host interactions and uncovers several N-interacting proteins that are new to PRRSV biology, but also highlights several key cellular machineries and pathways that are consistently exploited by members of *Nidovirales*. These findings may hold great promise for future host-oriented drug development.

## 2 Experimental Procedures

This study aimed to identify PRRSV-N interacting proteins. HEK293T cells were used as a cell model. N interacting proteins were enriched by performing GFP IPs using cell lysates from HEK293T cells overexpressing EGFP-PRRSV-N. Control IPs were performed using cell lysates from HEK293T cells overexpressing EGFP. Three biological replicates of EGFP-N and control IPs were performed. In addition, each biological replicate was injected twice, and data were combined before protein identification by MaxQuant to increase identifications. A threshold of 1% FDR at the peptide and protein level, which is the standard statistical criterion for the database search was used. Label-free quantification was performed by MaxQuant. In total, each pull-down data set consisted of three data points. Welch’s *t* test was used to compare N IP with control IP data sets. The details of the data analysis workflow are described in the following sections.

### 2.1 Cell Culture and Transfection

All the cell lines used in this study were maintained in Dulbecco’s modified Eagle’s medium (DMEM) supplemented with 10% fetal bovine serum (FBS) and incubated culture at 37 °C in the presence of 5% CO_2_. The N-terminally EGFP-tagged N expression vector (pMASIA-EGFP-N) was generated as previously described.^24^ For transfections, 10 cm dishes were seeded with 5 × 10^6^ HEK293T (ATCC CRL-3216) cells 1 day prior to lipofectamine 2000 transfection with 10 μg plasmid DNA coding for EGFP (pMASIA-EGFP) and EGFP-N. Each transfection reaction was performed independently in triplicate. At 48 h post-transfection, protein expression was examined under fluorescence microscope, harvested and subjected to EGFP IP.

### 2.2 EGFP Immunoprecipitation (EGFP IP)

EGFP and EGFP-N IPs were performed using 10 μL of a GFP trap (ChromoTek GmbH, Munich, Germany), which consists of a single-chain anti-GFP V_H_H conjugated to agarose beads. Cells were lysed at 4 °C for 1h on a rotary mixer in 1 mL lysis buffer (50 mM HEPES, pH 7.4, 150 mM NaCl, 1 mM EDTA, 10% glycerol, 1% Triton X-100 supplemented with protease inhibitor cocktail). The lysate was cleared by centrifugation at 14,000 rpm. The GFP-trap beads were equilibrated with ice-cold lysis buffer before adding to the cell lysate. The IPs were performed for 2 h at 4 °C on a rotary mixer. The beads were washed 7 times with 1 mL lysis buffer each time. After centrifugation and removal of the lysis buffer, the beads were resuspended in 2× Laemmli SDS protein loading buffer containing 100 mM DTT and boiled at 95 °C for 10 min to elute bound proteins for LC-MS/MS analysis.

### 2.3 Liquid-Chromatography Mass Spectrometry (LC-MS/MS) Analysis

IP samples were cleaned by running them into 10% acrylamide gels for approximately 1.5 cm. Subsequently, the gels were stained with Coomassie Blue R250. The gel lanes were then cut and subjected to in-gel tryptic digestion. Briefly, each gel lane was diced into smaller pieces and washed twice with 100  mM triethylammonium bicarbonate (TEAB) in 50% acetonitrile. The proteins were first reduced using 10  mM DTT in 100 mM TEAB at 56 °C for 1 h, and then alkylated using 55  mM iodoacetamide in 100 mM TEAB in the dark for 30 min at room temperature. After washing with 100  mM TEAB, followed by 100% acetonitrile for two cycles, the gel pieces were incubated with 1 μg trypsin (Promega) in 50 mM TEAB for 1 h on ice. Subsequently, the digestion was carried out overnight at 37 °C . The resulting peptides were lyophilized and stored at −80 °C until further use. These peptides were resuspended in 0.1% formic acid and subjected to LC-MS/MS.

Peptides were analyzed using a quadrupole orbitrap Q-Exactive Plus mass spectrometer (Thermo Fisher Scientific). Each sample was injected twice into the mass spectrometer to obtain two technical replicates. The analysis was carried out using reversed-phase liquid chromatography coupled to a nanoflow electrospray ion source (EASY-nLC 1000, Proxeon/Thermo Fisher Scientific) with a 250 mm C18 column (internal diameter: 75 μm). Peptide separation was performed at a flow rate of 300 nL/min over a 90 min gradient (5–20% acetonitrile in 60 min, 20–40% in 20 min, and 40–98% in 2 min). Survey full scan MS spectra (*m*/*z* 350 to 1400) of peptides were acquired in the Orbitrap at a resolution of 70,000. Higher energy collision dissociation was used as fragmentation mode. The MS instrument was operated in data- dependent acquisition (DDA) mode by automatically switching between MS and MS/MS acquisition. Signals with unknown charge states were excluded from fragmentation. The dynamic exclusion option was enabled (30 s). The ten most intense ions (charge state: *z* ≥ 2) were isolated and fragmented. The mass spectrometry proteomics data have been deposited to the ProteomeXchange Consortium via the PRIDE partner repository with the data set identifier PXD0044394.

### 2.4 Label-Free Quantification

All raw files were analyzed together using MaxQuant (version 1.5.3.30) with enabled label-free quantification (LFQ) option as described in ref 25. The two technical replicates of each biological replicate were combined before performing database search using MaxQuant. The derived peak list was searched using the Andromeda search engine against a concatenated database consisting of human UniProtKB/Swiss-Prot and TrEMBL (downloaded on 24/3/2016, containing 151,869 entries), EGFP sequence (GenBank accession number AAB08058.1), and N sequence from PRRSV strain 01NP1 (GenBank accession number DQ056373). A maximum of two missed cleavages was allowed, and the peptide length was set to a minimum of seven amino acids. Main search peptide tolerance was set at 4.5 ppm and fragment ion mass deviation of 0.5 Da was allowed. The fixed modification was carbamidomethylation of cysteines and variable modifications were oxidation of methionine and N-terminal acetylation of proteins. The false discovery rate (FDR) threshold for peptide and protein identifications was established at 1%. The “match between runs” feature was enabled with a match-time window of 0.7 min and an alignment-time window of 20 min. Label-free quantification of proteins was performed using the MaxLFQ algorithm integrated into MaxQuant.^26^ The “FastLFQ” option was enabled and the minimum ratio count was set at 2. For each pairwise peptide intensity comparison, it was required that at least one of the two peptides had been identified by MS/MS. LFQ intensities for respective protein groups were uploaded to Perseus. Reverse identifications, contaminants, and proteins labeled as “only identified by site” were removed from the data set. Subsequently, the LFQ intensities were log transformed. The three biological replicates were grouped, and identifications were filtered to retain proteins with valid values in all replicates for at least one IP group. Missing intensity values were imputed using random values drawn from the normal distribution of LFQ intensities. A Welch’s *t* test was performed comparing the N IP group to the control IP group. The differences between the logarithmic means in the EGFP-N IP group and control EGFP IP group were calculated and subsequently exponentially transformed to obtain the mean enrichment ratios. A protein was considered to be specifically enriched by N if its mean enrichment ratio was greater than or equal to 8 (*p* ≤ 0.02). Only the specifically enriched proteins that were identified with at least two peptides in all N IP replicates were considered highly confident N-interacting protein candidates.

### 2.5 Hypergeometric Gene Ontology (GO) Enrichment and Protein–Protein Interaction Network Analysis

The list of PRRSV N interacting proteins was analyzed using the DAVID tool (v.6.8, https://david.ncifcrf.gov/tools.jsp) to identify statistical enrichment of specific GO terms from the molecular function (MF), biological process (BP), and cellular component (CC). To establish protein–protein interactions among the identified interacting proteins, we utilized STRING v.11 (https://string-db.org).^27^ We seeded all proteins identified with an adjusted *p* ≤ 0.02 and enrichment ratio ≥ 8. Only the known interactions experimentally determined or from curated databases with a moderate combined interaction score ≥ 0.6 were used to construct the N interaction network. The N interaction map was constructed using Cytoscape.^28^

### 2.6 Validation of the Interactions between PRRSV N and Selected Protein Candidates

#### 2.6.1 Construction of HA-Tagged Cellular N-Interacting Proteins

RNA was extracted from PRRSV-permissive African green monkey (*Chlorocebus sabaeus*) MARC-145 cells (ATCC CRL-12231) and/or porcine (*Sus scrofa*) SK-6 cells^29^ using UPzol RNA isolation solution (Biotechrabbit GmbH). Subsequently, the first strand cDNA was synthesized from 2 μg of extracted RNA using RevertAid H minus first strand cDNA synthesis kit (Thermo Fisher Scientific). For the cloning of N-terminal HA-tagged csMYBBP1A (NCBI reference sequence: XM_008009916.1), csNCL (NCBI reference sequence: XM_007966617.1), csUPF3B (NCBI reference sequence: XM_008019435.1), csIGF2BP1 (NCBI reference sequence: XM_008013039.1), csG3BP1 (NCBI reference sequence: XM_008015063.1), csEIF2S1 (NCBI reference sequence: XM_007987056.1), csRFC4 (NCBI reference sequence: XM_008009481.1), csNOP2 (NCBI reference sequence: XM_007967352.2), ssABCF1 (NCBI reference sequence: XM_005653453.3), ssNSUN2 (NCBI reference sequence: XM_003359850.4), ssPPM1G (NCBI reference sequence: XM_005655254.3), and ssRTCB (NCBI reference sequence: NM_001122986.2). Kozak and HA-tag coding sequences were placed at the 5' end, immediately downstream of the restriction site of the forward primer. All the forward and reverse primers are shown in Table S1. PCR was performed using Phusion high-fidelity DNA polymerase (Thermo Fisher Scientific). PCR products were cloned into pcDNA3.1+ vector (Thermo Fisher Scientific). All the recombinant plasmids were transformed into *E. coli* DH5α. The candidate clones were first screened using a rapid size-screening method. DNA sequences of all the generated expression vectors were confirmed by sequence analysis (ATGC Co Ltd., Thailand).

#### 2.6.2 HA-Tag Coimmunoprecipitation (HA-tag Co-IP) Assay

Each 10 cm dish was seeded with 5 × 10^6^ HEK293 cells (ATCC CRL-1573) 1 day prior to calcium phosphate transfection with 5 μg of plasmid DNA encoding for EGFP-tagged N and 5 μg of each HA-tagged protein candidate. For control IP, cells were transfected with plasmid DNA encoding for PRRSV N and empty expression vector pcDNA3.1+. 48 h post-transfection, cells were collected and subjected to HA-tag Co-IP using either anti-HA agarose beads (28182, Thermo Scientific) or anti-HA nanobody-conjugated agarose beads (A310045, Antibodies.com). For all experiments, prior to bead addition, 1% of each supernatant was preserved and mixed with 5× Laemmli SDS protein loading buffer containing DTT for subsequent immunoblot analysis.

To disrupt RNA-mediated interactions, HA-tag Co-IP was performed in parallel in the presence or absence of RNase A. A total of 15 μL of beads was used per reaction, and IP was carried out as described above. After the first wash, samples designated for the RNase A condition underwent three consecutive washes with wash buffer containing RNase A (1.5 μg/mL). All washes were carried out at 4 °C for 5 min, except the second wash, which was performed at 25 °C for 30 min, then at 4 °C for 30 min. Subsequently, three additional washes were performed under standard conditions. Beads were subsequently transferred to a new tube. Bound proteins were eluted by resuspension in 2× Laemmli SDS loading buffer supplemented with 100 mM DTT and boiled at 95 °C for 10 min. Immune complexes were resolved by 10% SDS-PAGE, blotted onto PVDF membranes, and visualized with chemiluminescence after incubation with anti-HA (26183, Invitrogen) and anti-GFP (E-AB-20050, Elabscience) antibodies. GAPDH was used as a loading control.

### 2.7 Immunofluorescence Analysis (IFA)

For transfection, a well of a 24-well dish containing a coverslip was seeded with 35,000 HeLa cells (ATCC CRM-CCL-2) 1 day prior to Lipofectamine 3000 transfection with 0.25 μg of each plasmid DNA expressing EGFP-N and HA-tagged candidate protein. At 24 h post-transfection, cells were fixed with 4% paraformaldehyde in PBS for 20 min at RT, followed by three washes with PBS. Cells were permeabilized with 0.1% Triton X-100 for 15 min, followed by three washes with PBS. After blocking with 2.5% BSA in PBS for 30 min, cells were incubated with anti-HA tag antibody in 0.25% BSA in PBS overnight at RT, followed by three washes with PBS and incubation with TRITC-conjugated secondary antibody for 2 h at RT. The nuclei of the cells were stained using Hoechst (1 μg/mL) for 5 min, and after a final wash with PBS, the coverslips were mounted on microscope slides.

### 2.8 Generation of Stable Knockdown MARC-145 Cell Lines

Nontargeted (control) shRNA and shRNA targeting sequences for RTCB, MYBBP1A, and PPM1G were selected from a predesigned shRNA library (Sigma-Aldrich). The sense and antisense shRNA-coding oligonucleotides are listed in Table S2. These oligonucleotides were phosphorylated, annealed, and cloned into the *Hpa*I and XhoI sites of a modified version of the LentiLox 3.7 lentiviral plasmid (pLL3.7-puro),^30^ which contains a puromycin resistance gene for selection, and subsequently sequenced. The shRNA-expressing plasmids were packaged as lentiviruses by cotransfecting the packaging plasmid psPAX2 (Addgene #12260) and the envelope plasmid pMD2.G (Addgene #12259) into HEK293T cells.

Stable knockdown MARC-145 cell lines were generated by lentiviral transduction in the presence of 4 μg/mL of Polybrene, followed by puromycin selection at a concentration of 10 μg/mL until single colonies were observed. The knockdown efficiencies were assessed by Western blot analysis using antibodies against RTCB, MYBBP1A, or PPM1G (19809-1-AP, 15532-1-AP, 14524-1-AP, Proteintech). For each gene knockdown cell line, a nontargeted shRNA-expressing cell line with similar passage numbers was used as a control, and cell viability was assessed over five generations using the Trypan blue assay.

### 2.9 PRRSV Infection

Type 2 PRRSV strain 01NP1 ^31^ was kindly provided by Chulalongkorn University Veterinary Diagnostic Laboratory (CU-VDL), Bangkok, Thailand. The virus was propagated in MARC-145 cells, and the viral titer was determined by plaque assay. Briefly, viral supernatant was serially diluted, inoculated onto a monolayer of MARC-145 cells for 1 h, and overlaid with media containing 1.5% carboxymethylcellulose (Sigma-Aldrich) and 2% FBS. At 5 days postinfection, cells were fixed with 4% formaldehyde and stained with 1% crystal violet. The viral titer was calculated in plaque-forming units per milliliter (PFU/mL).

For viral infection, 1 × 10^6^ control or gene-knockdown cell lines were seeded in a well of a 6-well plate. The next day, 1 mL of virus stock, diluted in serum-free medium, was added to each well and incubated for 1 h at a multiplicity of infection (MOI) of 0.1. After incubation, the supernatant was removed, and the cells were washed twice with PBS. Then, 2 mL of culture medium containing 2% FBS was added, and cells were incubated for 48 h. Supernatants were collected, and viral titration was performed using plaque assay. Four replicates were conducted for each control and gene-knockdown cell line pair.

### 2.10 Structural Modeling of the PRRSV N–RTCB–RNA Ternary Assembly

To investigate whether RNA could act as a bridging scaffold between the PRRSV N protein and porcine RTCB, we performed structural modeling of a ternary complex consisting of N (NCBI reference sequence: AY745499.1), RTCB (NCBI reference sequence: NM_001122986.2), and an RNA molecule. Porcine spliced tRNA^Leu(CAA)^ (sequence derived from the precursor tRNA, NCBI reference sequence: NC_010449.5), a natural substrate of RTCB, was used as a representative RNA species in the modeling to illustrate RNA-mediated interactions. tRNA, together with Mn^2+^ ions, was used to predict three models: N–tRNA, RTCB–tRNA, and N–RTCB–tRNA complex structures via the AlphaFold 3 web server.^32^ Among the five models generated per system for N–RTCB–tRNA complex, the most reasonable conformation was selected and subjected to restrained molecular dynamics (MD) simulations using AMBER24 ^33^ to assess binding stability in solution, following our standard protocol.^34^ First, protonation states were assigned to all charged amino acid in the complex structures using the PDB 2PQR web server^35^ and subsequently solvated in a TIP3P water box extending 13 Å from the protein surface. Systems were neutralized by adding counterions. Each system was subjected to energy minimization using the steepest descent and conjugate gradient methods for 10,000 steps each. The Molecular Mechanics/Generalized Born Surface Area (MM/GBSA) method, implemented in AMBER using MMPBSA.py,^36^ was employed to calculate binding free energies (Δ*G*_bind_^MMGBSA^).

## 3 Results

### 3.1 Identification of PRRSV N Interacting Proteins

HEK293T cells were transfected with EGFP-N or empty EGFP expression vectors. To analyze the cellular interactions of PRRSV N, EGFP IP in combination with intensity-based, label-free quantitative proteomics was performed to enrich and identify N-interacting proteins (Figure 1A). Before collecting the cells for EGFP IP, the expression of EGFP and EGFP-N was confirmed by immunofluorescence microscopy. The analysis showed that EGFP-N localized to both the cytoplasm and the nucleus (Figure 1B). In the nucleus, the protein distributed throughout the nucleoplasm as well as to the nucleolus as previously reported in infected MARC-145 cells.^15^ At 48h post-transfection, lysates were prepared from the transfected cells and subjected to EGFP IP. SDS-PAGE analysis of the EGFP-IP immune complexes followed by Coomassie staining revealed the presence of EGFP and EGFP-N at the expected molecular masses, confirming the expression of these proteins and the successful enrichment of N (Figure 1C).

**Figure 1:**
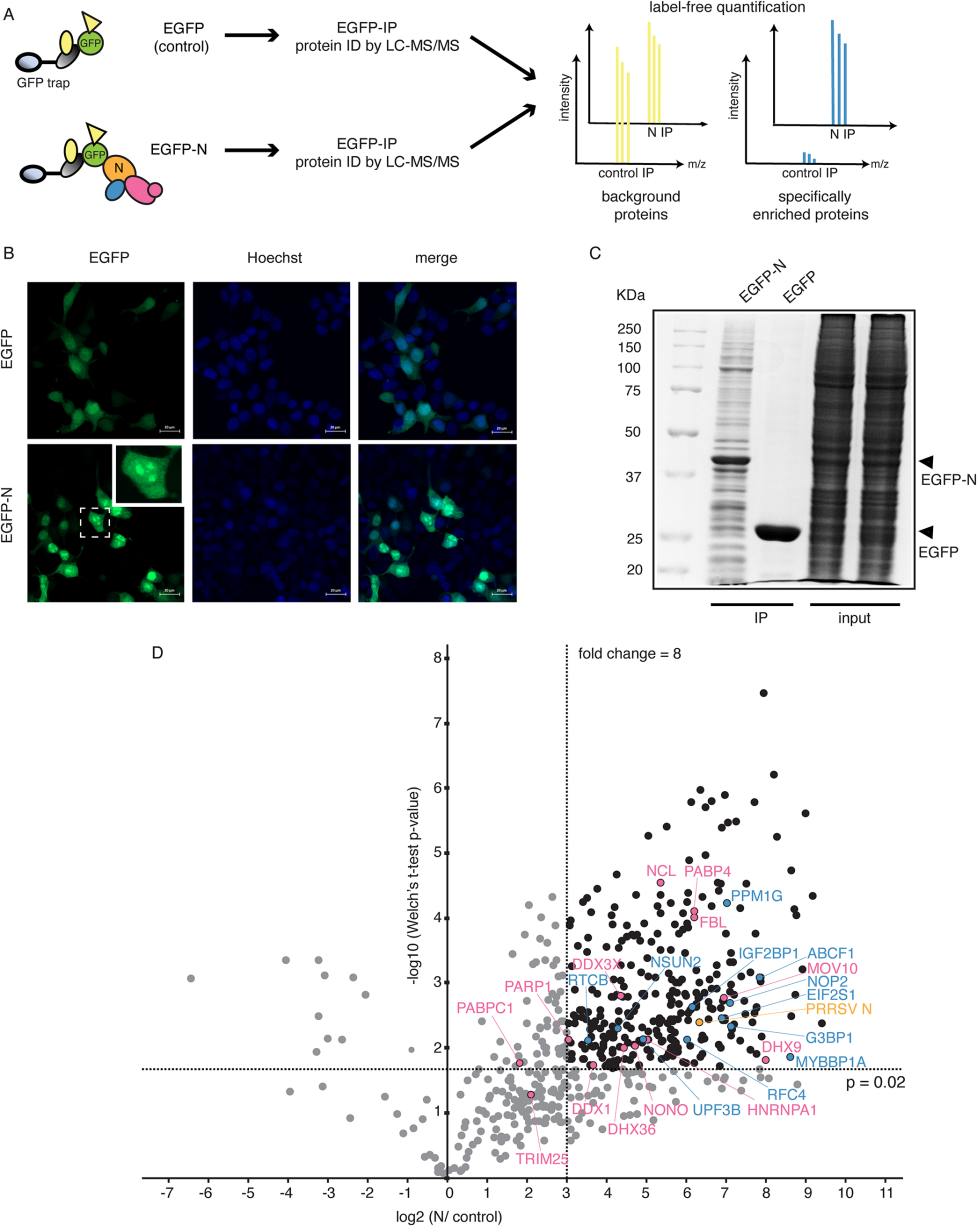
EGFP IP coupled with label-free quantitative mass spectrometry to identify PRRSV N interacting proteins. (A) Schematic representation of the methodology used in this study. EGFP-N together with its binding partners were enriched using GFP-trap beads. Enriched proteins were digested and analyzed using LC-MS/MS in a label-free fashion. (B) Expression and localization of EGFP and EGFP-N at 48 h after transfection. The nucleus was counterstained using Hoechst and is shown in blue, while EGFP and EGFP-N are represented in green. The inset shows an expanded view of the N localization. The scale bar represents 20 μm. (C) SDS-PAGE analysis of enriched proteins from control and EGFP-N IP. Black arrows indicate enriched EGFP and EGFP-N. (D) Volcano plot representing the analysis of PRRSV-N IP experiment. The log2 mean enrichment ratios of all proteins quantified in the PRRSV-N IP data set were plotted against the corresponding -log10 *p*-values. Proteins passing the thresholds of *p*-value below 0.02 (≥1.7 on a negative log10 scale) and mean enrichment ratio ≥ 8 (3 on a log2 scale) were treated as PRRSV-N interacting protein candidates and are represented in black. Pink circles highlight known PRRSV-N interacting proteins or interactions verified in previous studies,^37–45^ while blue circles highlight interactions verified in this study.

A label-free quantification (LFQ) approach was performed to compare the EGFP-N to the control EGFP experiments, and a stringent cutoff threshold was set to eliminate nonspecific binding proteins that arise due to binding to either EGFP moiety, the antibody, or the matrix. The list of all protein groups identified and peptides assigned to each protein are provided in Tables S3 and S4 respectively. Proteins exhibiting enrichment ratios greater than or equal to 8 fold with *p*-values less than or equal to 0.02 were considered PRRSV N-interacting protein candidates (Figure 1D).

A total of 301 proteins were identified as PRRSV N-interacting candidates (Figure 2, Table S5). To assess the validity of these candidates, we compared our data with previous PRRSV N interactomics studies, which reported a total of 362 PRRSV N-interacting proteins.^37–39^ Of these, 82 proteins overlapped with our findings (Figure 2, thick border), representing high-confidence interactions consistently detected across different AP-MS approaches. The remaining 219 proteins (∼70%) were newly identified in this study, expanding the PRRSV N interaction landscape.

**Figure 2:**
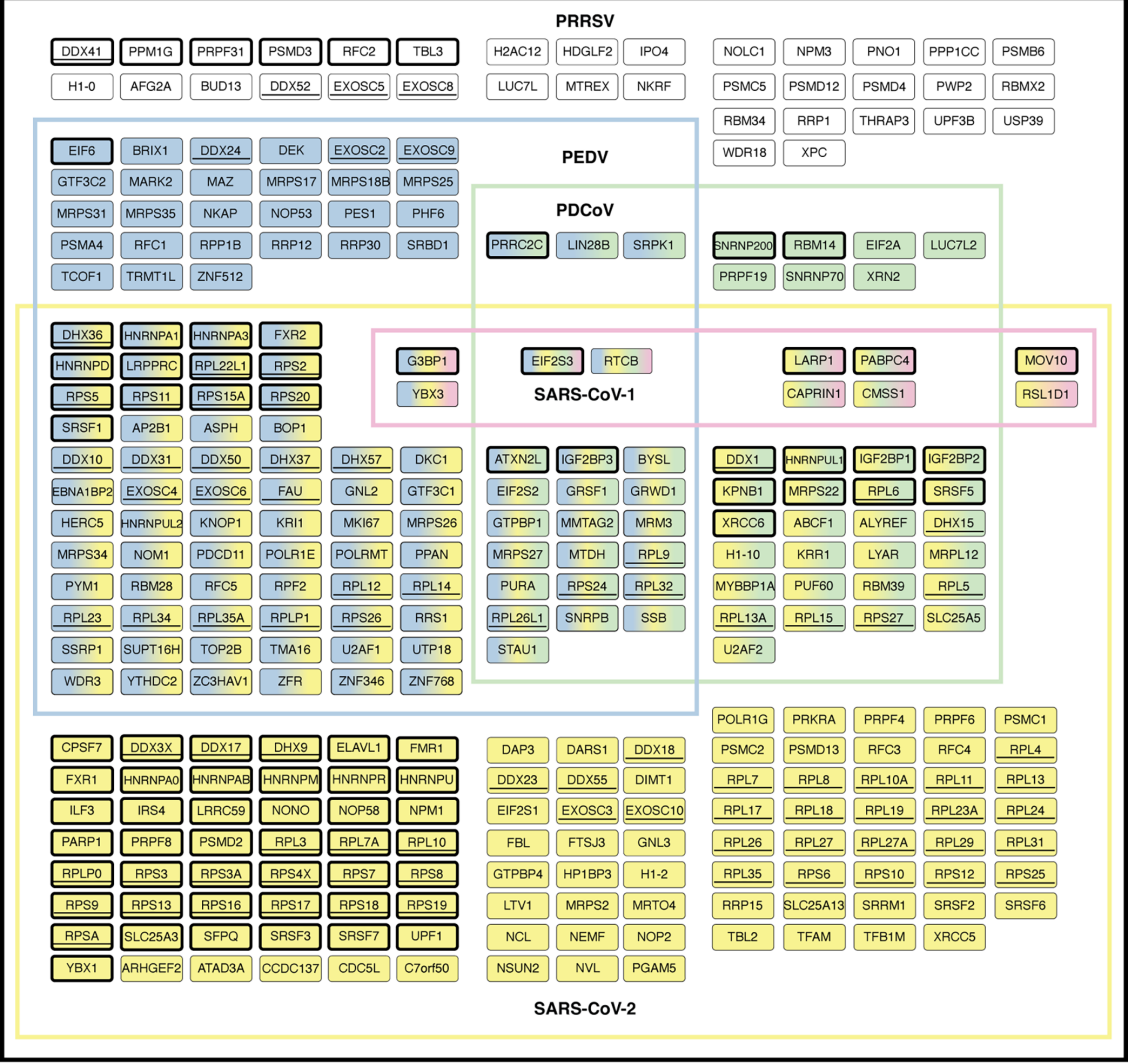
Cellular proteins identified as PRRSV N-interacting protein candidates using EGFP IP-MS. A total of 301 protein candidates identified in this study are presented by their gene names. The protein candidates that have been identified as PRRSV-N interacting proteins in previous studies are indicated using thick, black borders. Overlapping N interactors between PRRSV and PEDV are highlighted in blue, while those shared between PRRSV and PDCoV are shown in green. Overlapping N interactors between PRRSV and SARS-CoV-1 and SARS-CoV-2 are indicated in yellow and pink, respectively. Multicolored boxes represent PRRSV N-interacting protein candidates shared with more than one virus. N interacting protein candidates uniquely identified in our study are represented by white boxes. Proteins belonging to the RNA helicase (DDX, DHX) gene families, ribosomal protein (RPL, RPS) gene families, and RNA exosome (EXOSC) gene family are highlighted using underlining.

Related virus families often exploit common host factors and pathways, and conserved virus-host interactions may highlight key host factors essential for viral replication. To further explore the functional relevance of our N interactome, we compared our data set with high-throughput interactomics studies on *Nidovirales* members. Notably, two independent studies identified 426 and 243 N-interacting proteins from porcine enteric coronaviruses, porcine epidemic diarrhea virus (PEDV) and porcine deltacoronavirus (PDCoV),^46,47^ whose natural coinfection is commonly observed in diarrheal pigs.^48,49^ Additionally, we compiled 68 unique interactors of SARS-CoV-1 N from two independent studies^50,51^ and 998 protein components of the SARS-CoV-2-N molecular landscape from 12 independent studies.^50,52–62^

Through our comparative analysis, we identified 266 shared N-host interactions, accounting for ∼90% of the interactions identified in our study. The analysis revealed a notable overlap among the porcine *Nidoviruses*, with 60 shared interactions with PDCoV and 117 with PEDV. Notably, there was a significant overlap of 229 interactions with SARS-CoV-2, although this could partly be attributed to the larger data set used for comparison. In contrast, only 10 shared interactions were identified between PRRSV and SARS-CoV-1, likely due to the smaller dataset of SARS-CoV-1 N- interacting proteins.

Our comparative study identified eukaryotic translation initiation factor 2 subunit 3 (eIF2-γ, EIF2S3) and tRNA-splicing ligase Rtcb homologue (RTCB) as host factors targeted by N proteins across all five *Nidovirales* members. EIF2S3, together with the α and β subunits (EIF2S1 and EIF2S2), forms the eukaryotic initiation factor 2 complex (eIF2), which facilitates initiator tRNA binding and transfer to the ribosome during the early translation. RTCB, the catalytic subunit of the tRNA ligase complex (tRNA-LC), is consistently identified as a common target of viral factors across various ssRNA virus families.^46,47,50,52,53,60,63–70^ Our findings further highlight RTCB’s potential role in Nidovirus infection, warranting further investigation.

In addition to eIF2 and tRNA-LC components, several proteins with known roles in Nidovirus infections, including helicase MOV-10 (MOV10),^43,71^ polyadenylate-binding protein 4 (PABPC4),^72^ La-related protein 1 (LARP1),^67,73^ and Y-box-binding protein 3 (YBX3),^74,75^ were among those identified as strongly conserved across multiple Nidovirus species. Consistent with the well-established association between coronavirus N proteins and stress granules (SGs),^53,54,76–78^ SG-related proteins such as ataxin-2-like protein (ATXN2L), caprin-1 (CAPRIN1), Ras GTPase-activating protein-binding protein 1 (G3BP1), and the double-stranded RNA-binding protein Staufen homologue 1 (STAU1) were also widely conserved N-interacting proteins across *Nidovirales* members. Notably, we observed a significant enrichment of DEAD-box and DExH-box RNA helicases (DDX and DHX) gene families, as well as ribosomal proteins from the large and small subunit (RPL and RPS) gene families (Figure 2, underlined), suggesting that N functions as a central hub for RNA helicase and ribosome interactions. Additionally, RNA exosome complex (EXOSC) subunits were shared among SARS-CoV-2, PEDV, and PRRSV (Figure 2, underlined), indicating a conserved viral strategy for engaging the RNA exosome, which is crucial for 3′-to-5′ RNA degradation and viral RNA decay.^79–81^

### 3.2 Features of the Cellular Proteins Targeted by PRRSV N

Gene Ontology (GO) enrichment analysis was performed to characterize the molecular functions (MF), biological processes (BP), and cellular components (CC) associated with the PRRSV-N interactome (Figure 3A,B, Table S6). The analysis revealed a strong enrichment of RNA-binding proteins (RBPs) (Figure 3A), suggesting that PRRSV N potentially modulates RNA-related processes within infected cells. Given that RBPs often copurify with RNA, some identified N-host interactions may be RNA mediated.

**Figure 3:**
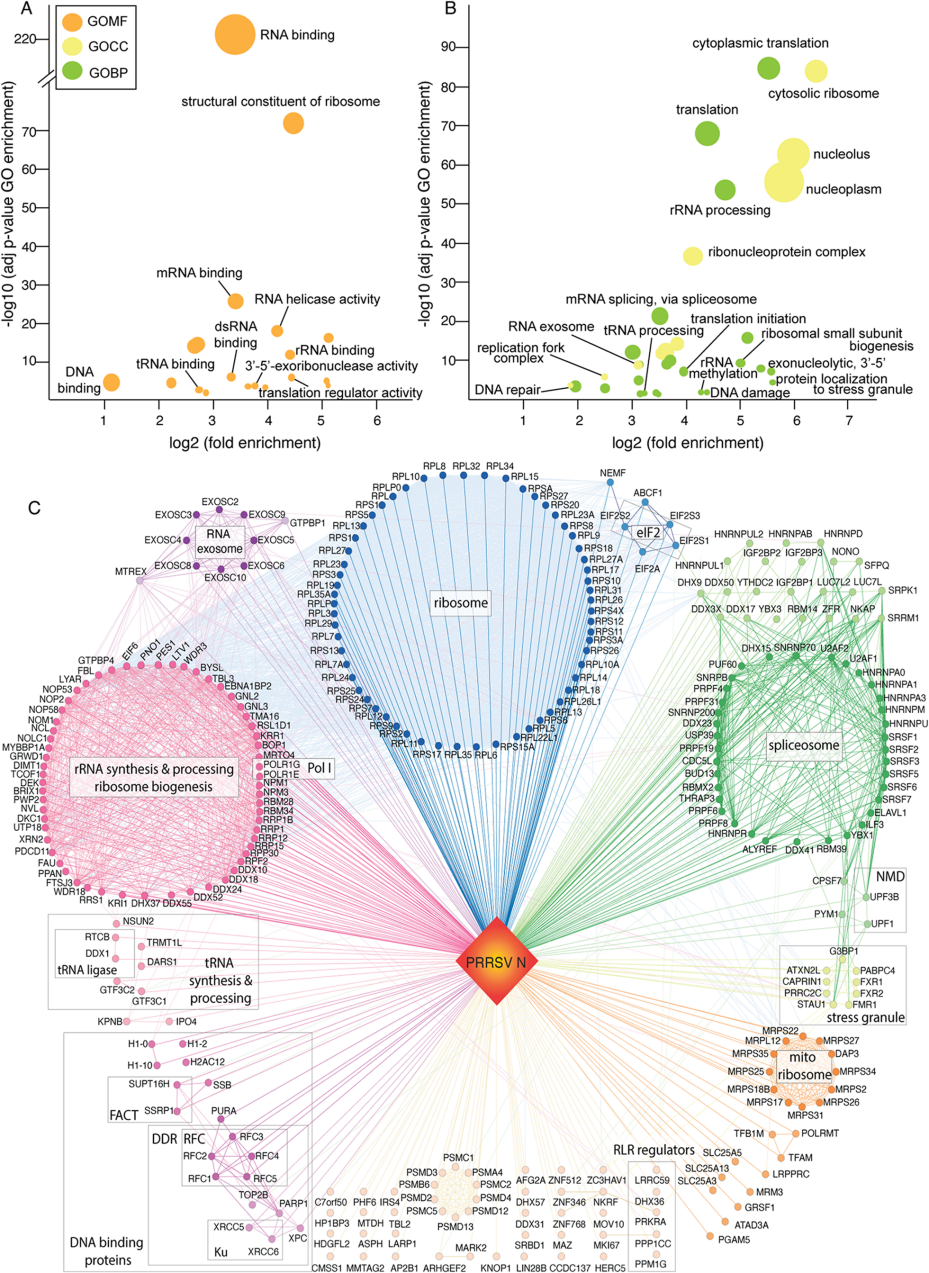
Features of the PRRSV N interactome. Bubble visualization of (A) GO molecular functions (GOMF), and (B) cellular components (GOCC) and biological processes (GOBP) enriched in the interactome. Circles represent enriched GO terms, and circle size corresponds to the number of proteins assigned to each term. (C) Protein–protein interaction (PPI) network generated by Cytoscape using 301 PRRSV N-host PPIs and 3,447 curated host PPIs from the STRING database. Nodes represent cellular proteins identified as PRRSV-N interacting protein candidates, and edges represent PPIs. The interactome is displayed as colored subnetworks.

As expected, GO terms related to ribosomes and translation were significantly enriched, alongside BP terms linked to ribosome biogenesis (Figure 3B), including rRNA processing, methylation and ribosome subunit assembly. These processes form a highly interconnected subnetwork (Figure 3C), and align with the predominant localization of N in the nucleolus, a key site for ribosome biogenesis. Overall, these findings highlight the close association of PRRSV N with ribosome functions and suggests its potential role in regulating ribosome biogenesis, which will be further discussed below.

STRING analysis identified 3,447 protein-protein interactions (PPIs), forming several main clusters that align with the enriched GOBP and GOCC terms (Figure 3B,C). These clusters include the tRNA synthesis and processing pathway, the eIF2 complex, and the spliceosome. Notably, enrichment of spliceosomal proteins and associated factors was also observed in a previous study of the PRRSV N interactome in porcine alveolar macrophages (PAMs).^39^ This finding suggests that PRRSV N may hijack the host splicing machinery, a hypothesis further supported by evidence that PRRSV rewires alternative splicing events in infected animals.^59^ Interestingly, the analysis also revealed an enrichment of dsRNA-binding proteins that regulate the viral RNA-sensing pathway of innate immunity, including ATP-dependent DNA/RNA helicase DHX36 ^82^ and interferon-inducible double-stranded RNA-dependent protein kinase activator A^83^ (PRKRA). Additionally, components of cell-intrinsic antiviral systems, including nonsense-mediated decay (NMD),^84^ stress granules,^85^ and RNA exosome^79–81^ were also enriched (Figure 3C).

Consistent with the enrichment of the DNA-binding GO term, PPI network analysis identified a subnetwork of nucleosome-remodeling proteins, including linker histone H1 and the FAcilitates Chromatin Transcription (FACT) complex, as well as Replication Factor C (RFC), the DNA replication clamp loader, and the Ku70 (XRCC6)/Ku80 (XRCC5) heterodimer of the DNA-dependent protein kinase (DNA-PK) complex. These factors facilitate DNA repair within the DNA damage response (DDR) pathway (Figure 3C). The presence of these nuclear proteins raises the possibility that the nuclear pool of PRRSV N plays specific roles in regulating chromatin structure and the DDR pathway, which will be discussed in the next section.

### 3.3 Validation of the Interactions between PRRSV N and Selected Protein Candidates by HA-tag Co-IP and Immunolocalization Assays

To confirm the interactions identified in the proteomic screen, we performed reverse HA-tag Co-IP assays using a subset of host proteins selected from different clusters in the STRING analysis (Figure 4, Figure S1). Interactions between the RNA-binding PRRSV N and these candidates were consistently detected under standard conditions. To assess whether these interactions were mediated by RNA, parallel Co-IP experiments were performed in the presence of RNase A. The candidate genes encoding these host proteins were cloned from either MARC-145 cDNAs or porcine cDNAs, as specified in the method section. The Co-IP assay was carried out in HEK293 cells where EGFP-N and each respective HA-tagged protein candidate were overexpressed. Subsequently, we investigated the colocalization pattern of PRRSV N and these protein candidates in HeLa cells (Figure 5).

**Figure 4:**
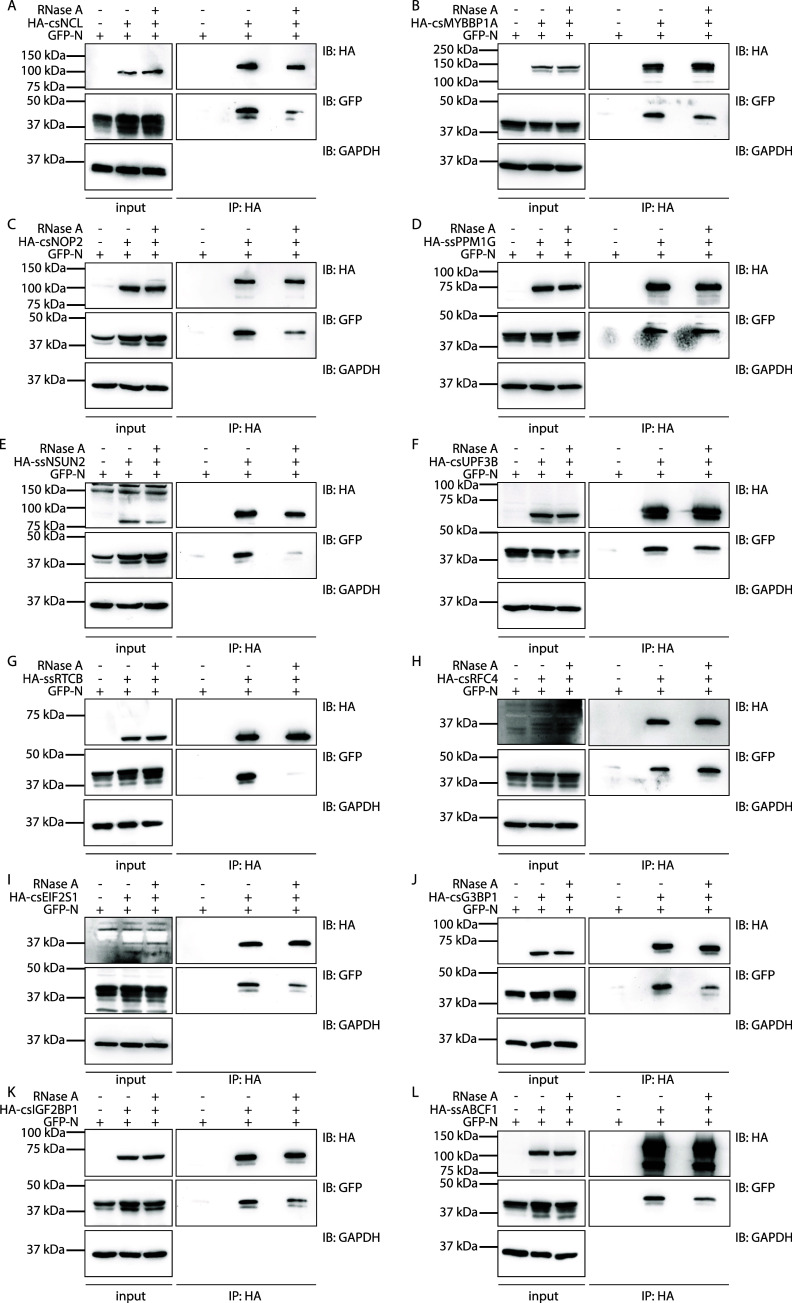
Validation of PRRSV N interactions with selected host proteins. HEK293 cells were transfected with GFP-tagged PRRSV N and HA-tagged candidate expression vectors or empty vectors (negative control) as indicated. Parallel Co-IP assays were performed in the presence or absence of RNase A to assess the RNA dependency of the interactions. cs represents candidate genes cloned from cDNAs of the PRRSV-permissive African green monkey (*Chlorocebus sabaeus*) cell line MARC-145, while ss represents candidate genes cloned from porcine (*Sus scrofa*) cDNAs. Cell extracts were subjected to HA-tag IP and immunoblotting using HA, GFP, and GAPDH (loading control) antibodies as indicated.

**Figure 5:**
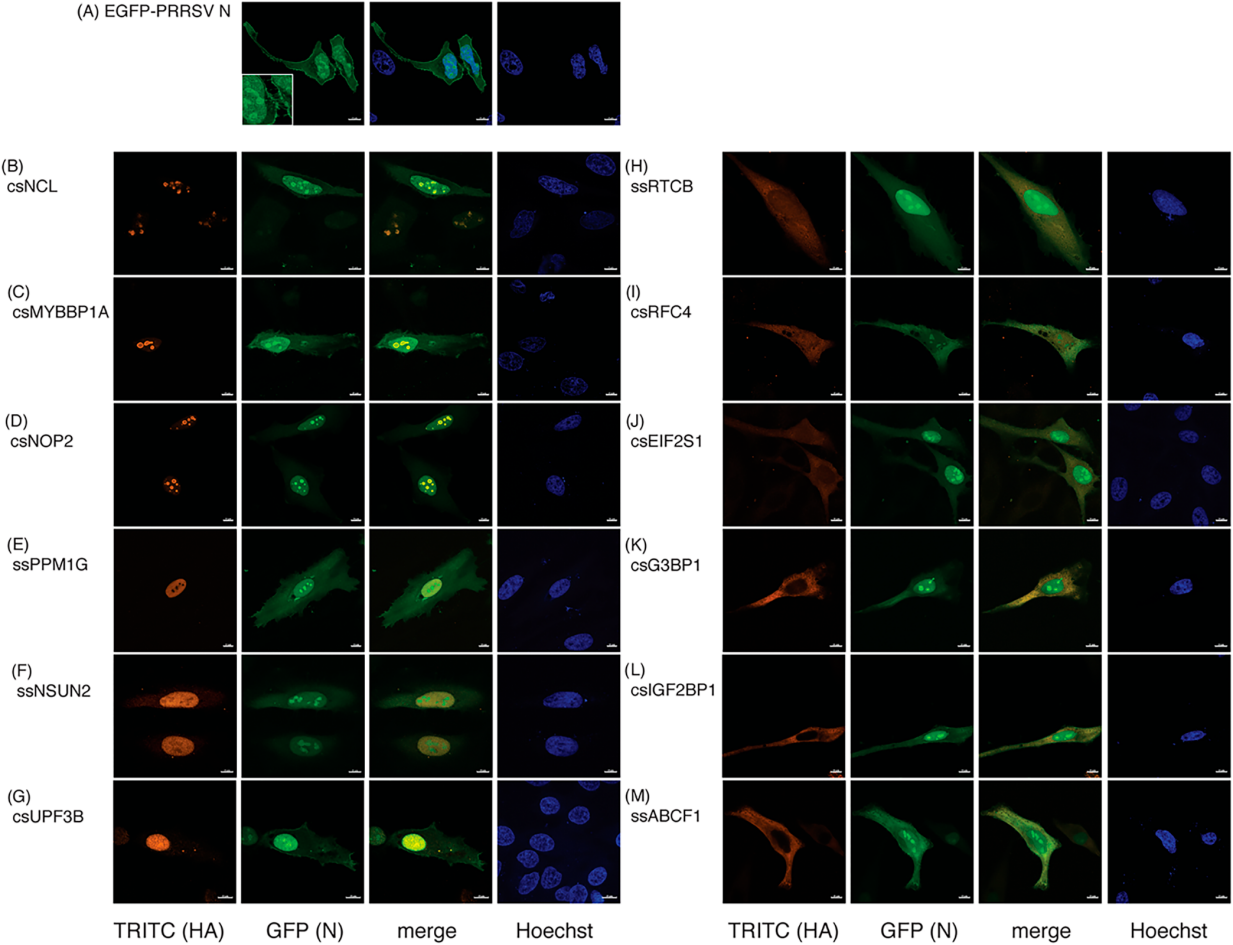
Colocalization immunofluorescence analysis of the validated HA-tagged N interacting proteins in HeLa cells. (A) EGFP-tagged PRRSV N and (B–M) EGFP-N and HA-tagged candidate expression vectors as indicated. At 24 h post-transfection, cells were stained with anti-HA antibody (red). Nuclei were counterstained Hoechst (blue). The yellow color represents an overlay of EGFP-N (green) and HA-tagged protein candidate (red) signals. The scale bar represents 10 μm.

We first examined the previously reported interaction between nucleolin (NCL) and PRRSV N protein^40^ (Figure 4A), as well as the association of PRRSV N with the well-characterized coronavirus N-interacting protein, G3BP1 ^53,54,76–78^ (Figure 4J). Both interactions were markedly reduced following RNase A treatment, indicating an RNA-dependent nature. We next examined two nucleolar proteins involved in rRNA biogenesis: Myb-binding protein 1A (MYBBP1A), a regulator of rDNA transcription, and 28S rRNA (cytosine-C(5))-methyltransferase (NOP2). Both interactions were RNA-mediated (Figure 4B,C), and robust colocalization with PRRSV N was observed in the nucleolus (Figure 5C,D). We further investigated host factors involved in tRNA metabolism. The interactions of PRRSV N with RNA cytosine C(5)-methyltransferase (NSUN2) and the tRNA ligase RTCB were almost completely abolished following RNase A treatment (Figure 4E,G), strongly indicating RNA-dependent associations. Immunofluorescence analysis showed that NSUN2, predominantly localized in the nucleus with minor cytosolic distribution, and RTCB, broadly distributed across the nucleoplasm and cytoplasm, both exhibited partial colocalization with N (Figure 5F,H)

Among host factors associated with mRNA translation, HA-tag Co-IP assays confirmed interactions of N with regulator of nonsense transcripts 3B (UPF3B), the α subunit of eIF2 (EIF2S1), as well as insulin-like growth factor 2 mRNA-binding protein 1 (IGF2BP1) and ATP-binding cassette subfamily F member 1 (ABCF1) (Figure 4F,I,K,L). Interactions with N were generally markedly reduced upon RNase A treatment, whereas UPF3B showed partial reduction. Consistent with its predominantly nuclear distribution, UPF3B strongly colocalized with PRRSV N in the nucleus, and we also observed colocalization of UPF3B and N within cytosolic condensates (Figure 5G). EIF2S1 localized primarily to the cytosol, while IGF2BP1 and ABCF1 exhibited a diffuse cytoplasmic distribution. All three proteins showed colocalization with cytosolic N (Figure 5J,L,M). In contrast, two host factors, protein phosphatase 1G (PPM1G) and subunit four (RFC4) of the DNA replication clamp loader RFC complex, interacted with PRRSV N in an RNA-independent manner, as their associations remained unchanged following RNase A treatment (Figure 4D,H). PPM1G was predominantly nuclear and exhibited strong colocalization with N (Figure 5E), whereas RFC4 displayed a broad nuclear and cytoplasmic distribution with partial colocalization with N (Figure 5I).

Notably, our immunolocalization assay revealed a distinct pool of PRRSV N protein at the cell surface and within membrane protrusions in cells expressing EGFP-N alone or coexpressing HA-tagged protein candidates. Representative images show robust N protein expression localized prominently at these regions (Figure 5A). Similar surface-localized features have been observed in other RNA viruses, including betacoronaviruses, where viral proteins act as chemokine modulators and targets for antibody-based immunity.^86–88^ This study provides the first evidence of PRRSV N protein localization to the cell surface, suggesting an additional subcellular distribution that may contribute to the immunosuppressive properties of PRRSV N.

### 3.4 Knockdown of RTCB or MYBBP1A Impairs PRRSV Replication in MARC-145 Cells

RTCB, MYBBP1A, and PPM1G were selected to study their functional roles in PRRSV infection. First, we generated MARC-145 cell lines with RTCB, MYBBP1A, or PPM1G gene knockdown. The knockdown efficiencies of these cell lines, compared to those of their control cell lines, were validated by Western blot analysis (Figure 6A). The growth kinetics of PRRSV in MARC-145 cells showed that the virus was first detected in the supernatants at 12 h postinfection (hpi) and plateaued at 48 hpi (Figure 6B), suggesting 48 hpi as the optimal time point to study the effect of gene knockdown on PRRSV infection. Each gene knockdown cell lines, along with its control cell line, was infected with PRRSV for 48 h at an MOI of 0.1. Thereafter, the cell culture media were collected and subjected to plaque assays to determine viral titers. As shown in Figure 6C, knockdown of RTCB or MYBBP1A led to a significant reduction in viral production, with decreases of 98- and 84-fold, respectively. In contrast, no notable change in PRRSV titers was observed in PPM1G knockdown cells compared to control cells, suggesting that PPM1G knockdown does not impact virus production.

**Figure 6:**
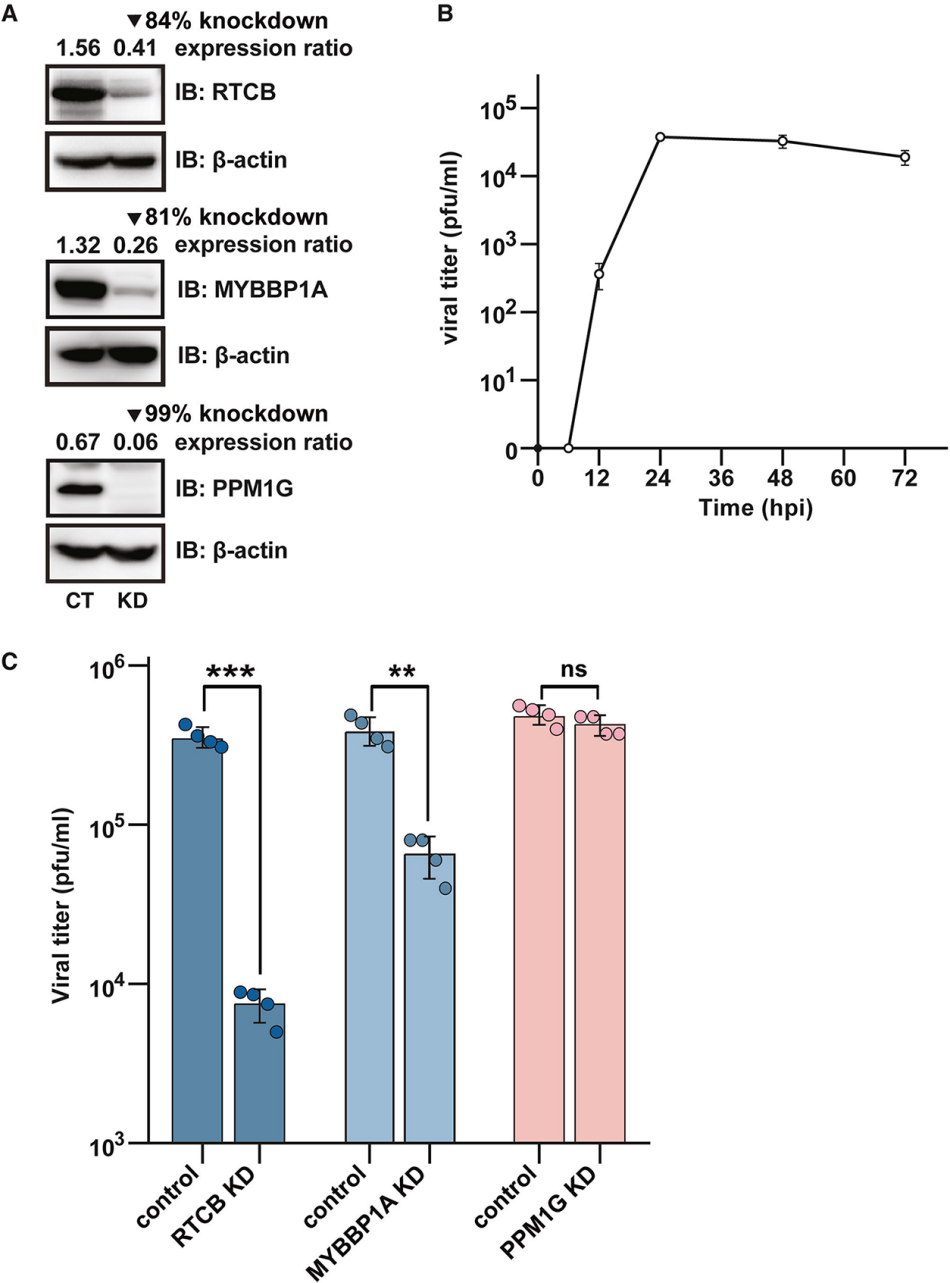
Knockdown of RTCB or MYBBP1A reduces PRRSV viral production in MARC-145 cells. (A) Stable RTCB-, MYBBP1A- or PPM1G-knockdown cell lines were generated using lentiviral shRNAs in MARC-145 cells. The expression levels of the target protein were examined by Western blot analysis. Protein expression ratios were calculated by normalizing to β-actin, and the percent knockdown was determined by comparing the expression ratios of the gene knockdown cell lines to those of the control cell lines. (B) Growth kinetics of PRRSV in MARC-145 cells. The results are expressed as mean ± SD for experiments performed in triplicate. (C) Comparison of PRRSV titers in culture supernatants of stable knockdown MARC-145 cell lines with their corresponding control cell lines at 48 hpi. The results are expressed as mean ± SD for experiments performed in four biological replicates. ***P < 0.001; **P < 0.01, ns, nonsignificant. Two-tailed Student’s *t*-test.

### 3.5 Structural Modeling of N–RTCB–RNA Interactions

Given that the interaction between RTCB and N was strongly RNA-dependent, we next explored whether RNA could act as a bridging scaffold between the two proteins. To this end, we modeled the ternary assembly of PRRSV N, porcine RTCB, and a representative RNA species, porcine spliced tRNA^Leu(CAA)^. Across the highest-scoring models, the N-terminal region of N consistently engaged the RNA, consistent with prior evidence of N-terminal RNA recognition.^14^ Remarkably, even without direct restraints between RTCB and the RNA, the N-bound tRNA spontaneously contacted RTCB, extending into its positively charged RNA-binding groove. This arrangement produced a bridged architecture in which the RNA served as the principal interface between N and RTCB (Figures 7 and S3). These findings suggest a chaperone-like role for the N protein at its N-terminal region (residues 1–57), facilitating delivery of RNA substrates to RTCB and promoting ternary complex formation. Variations in Δ*G*_bind_^MMGBSA^ rankings indicate that the identity of the RNA may influence the relative stability of these assemblies, although the calculations were used here for comparative purposes, rather than absolute binding free energy estimation. Beyond the ternary assembly, we also observed that in both the N–tRNA and RTCB–tRNA binary complexes, the RNA can bind independently to N or RTCB (Figures 7 and S3). The binding patterns in these binary models resemble those in the ternary complex, with the tRNA inserting into the conserved binding pocket of RTCB, while being partially stabilized by the N-terminal region of N (Figures 7 and S3).

**Figure 7:**
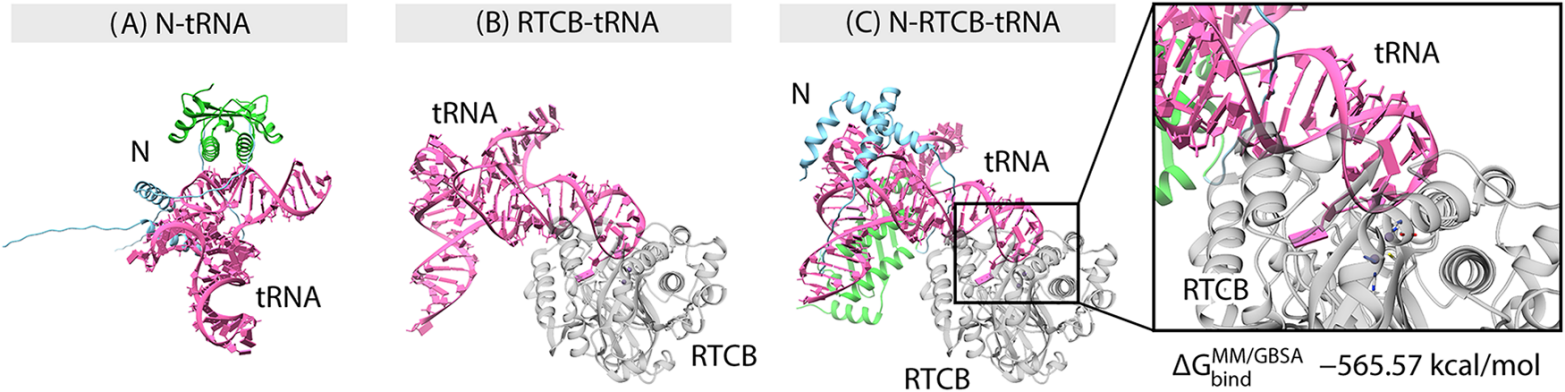
Predicted binary and ternary complexes of the PRRSV N protein, RTCB, and tRNA. (A) Binary complex of the PRRSV N protein with tRNA (N–tRNA). (B) Binary complex of RTCB with tRNA (RTCB–tRNA). (C) Ternary complex of the PRRSV N protein, RTCB, and tRNA (N–RTCB–tRNA). The inset highlights the close association of tRNA with RTCB, and the binding free energy estimated using the MM/GBSA method supported the high stability of the ternary complex. The N-terminal and C-terminal regions of the PRRSV N protein are depicted in green and blue, respectively; tRNA is shown in pink, and RTCB is shown in gray.

## 4 Discussion

PRRSV continues to pose challenges within the pig industry due to the continuous emergence of genetically diverse strains, driven by its extremely high mutation rate. The primary countermeasure currently employed to control this heterologous virus involves the use of modified live or attenuated vaccines. However, challenges persist, as these commercial vaccines are unable to confer broad protection. Compounding the issue, it has been observed that several vaccine strains can undergo genetic recombination with field strains, leading to the expansion of a more complex viral gene pool and presenting further difficulties in virus control.^89–92^

Given these considerations, there emerges the need to develop alternative approaches that center around host factors that are indispensable during the course of infection, given their genetic stability in comparison to viral factors. Within this context, PRRSV N positions as a strong candidate to initiate the exploration of host dependency factors. The protein is notable as the most abundant viral protein produced in infected cells with high immunogenicity. It serves as the sole structural component that binds to the genomic RNA, orchestrating the packaging of the viral genome. Recent research has shed light on a broader scope of N functionalities, unveiling its involvement in additional key roles spanning virus replication, pathogenesis, and host responses.

Currently, our understanding of the extra-genome packaging roles of the N protein primarily stems from research on viruses within the *Coronaviridae* family, which are the most extensively studied members of the *Nidovirales*. However, when it comes to the precise mechanisms and diverse functions of the Arterivirus N protein, especially its interaction landscape with host factors, our knowledge is relatively limited.

To bridge this gap, we undertook a comprehensive analysis of the PRRSV N protein, aiming to shed light on the landscape of PRRSV-host interactions. Our findings not only expand the current landscape of PRRSV N interactome, but also highlight several key cellular machineries and pathways that are consistently exploited by members of *Nidovirales*. It is noteworthy that a significant number of the proteins identified in our study (266 candidates or ∼90%) have been previously reported as interacting with coronavirus N proteins or as essential coronavirus host factors. This substantial overlap strongly suggests that these conserved interactions are of utmost importance for the replication of Nidoviruses and may hold great promise for future host-oriented drug development. In the following sections, we will delve into selected PRRSV N-interacting factors and pathways in greater detail.

### 4.1 PRRSV N Interaction with the tRNA Ligase Complex (tRNA-LC)

Among the cellular proteins copurified with the PRRSV N protein are RTCB and the DEAD-box helicase DDX1, which are the two core components of the five-subunit tRNA-LC. The catalytic subunit RTCB is an atypical RNA ligase that mediates the joining of 5′–OH termini to 2′,3′-cyclic phosphate termini. This termini pair is generated by specific endoribonucleases such as tRNA splicing endonuclease, RNase A, and RNase L.^93,94^ Notably, the serine/threonine-protein kinase/endoribonuclease IRE1-mediated splicing of X-box-binding protein 1 (XBP1) mRNA during the unfolded protein response also relies on tRNA-LC.^95^

The tRNA-LC is a known host factor for several ssRNA viruses, including influenza A virus (IAV),^65^ Sindbis virus (SINV),^63^ Dengue,^64^ Zika,^64^ and coronaviruses such as SARS-CoV-1,^50^ SARS-CoV-2,^50,52,53,60,62,66–69^ PEDV,^46^ PDCoV^47^ and avian bronchitis virus (IBV).^70^ It has been identified as a viral RNA (vRNA)-binding^66–69^ or N-interacting complex^46,47,50,52,53,60,62,70^ (Table S5) in these viruses. In our study, RTCB knockdown significantly reduced PRRSV viral production, highlighting the tRNA-LC as a host dependency factor for PRRSV. Consistently, knockdown of tRNA-LC components also impaired SARS-CoV-2 RNA and N protein levels in infected cells.^66^ Collectively, these lines of evidence position the tRNA-LC as a key host factor with a master regulatory role in Nidovirus infections.

While the precise mechanism remains unclear, studies show that in SARS-CoV-2 infected cells, the tRNA-LC relocates from the nucleus to cytoplasmic foci where viral RNA accumulates,^66^ suggesting a direct role in viral genome processing. Since ssRNA viruses are vulnerable to RNA damage induced by the 2′,5′-oligoadenylate synthetase (OAS)-RNase L pathway, which cleaves vRNA upon activation.^96,97^ Notably, studies have reported the upregulation of OAS-RNase L pathway genes in PRRSV-infected cells and animals.^98,99^ Given the ability of the tRNA-LC to repair RNase L-induced RNA breaks, it is plausible that the interaction between PRRSV N protein, vRNA, and tRNA-LC facilitates the repair of these breaks, thereby enhancing viral genome stability.

Our data further refine the role of RTCB in PRRSV infection. Co-immunoprecipitation experiments revealed that the interaction between N and RTCB is strongly RNA-dependent. Structural modeling suggests that N facilitates RNA-mediated bridging to the RTCB binding groove, guiding RNA substrates to promote ternary complex formation. Because RTCB has previously been reported to bind viral genomic RNA,^66–69^ it is plausible that similar RNA-dependent scaffolding could occur during infection. Such model is consistent with the broader concept that ssRNA viruses exploit the tRNA ligase to stabilize their genomes during infection.

Beyond Nidoviruses, tRNA-LC has been shown to promote IAV replication through immune modulation.^100^ Depletion of RTCB led to a significant increase in type I and type III IFNs and proinflammatory cytokines in response to infection. Mechanistically, RTCB disrupts the DDX21-DDX1 interaction, which otherwise promotes IFN and downstream IFN-stimulated genes.^100^

Overall, this suggests that tRNA-LC components may have a broader impact on viral pathogenesis. Future research should focus on elucidating the precise molecular mechanisms by which tRNA-LC supports Nidovirus replication.

### 4.2 PRRSV N Hijacks Nucleolus, Targeting the rRNA Pathway

Despite most RNA viruses relying on cytoplasmic replication, growing evidence suggests that many target nuclear subcompartments to facilitate their replication. The Nidovirus N protein is a well-characterized examples of a viral factor undergoing nucleocytoplasmic shuttling, with its nuclear and nucleolar localization signals extensively studied.^15,16,101–104^ Notably, the nuclear pool of N has been linked to severe pathogenesis in coronaviruses,^105^ while mutations preventing nuclear localization have been shown to impair PRRSV replication and pathogenesis in infected animals.^18,19^

Within the nucleolus, critical processes in rRNA biogenesis occur, including rRNA transcription, processing, and assembly with ribosomal proteins to form ribosomal subunits. Our study revealed a significant enrichment of host proteins involved in rRNA biogenesis (Figure 3C), including RNA polymerase I (Pol I) subunits POLR1G and POLR1E, as well as rDNA transcription regulators such as treacle protein (TCOF1),^106^ nucleolar and coiled-body phosphoprotein 1 (NOLC),^107^ NCL,^108^ nucleophosmin (NPM1),^109^ MYBBP1A,^110^ periodic tryptophan protein 1 homologue (PWP),^111^ and cell growth-regulating nucleolar protein (LYAR).^112^ We also identified multiple pre-rRNA processing and maturation factors, including the PeBoW complex components pescadillo homologue (PES1) and ribosome biogenesis protein BOP1,^113^ the 5′-3′ exoribonuclease XRN2,^114^ and the nuclear RNA exosome complex.^115^ Furthermore, rRNA modification machinery was represented by H/ACA box and C/D box small nucleolar ribonucleoproteins (snoRNPs), including H/ACA ribonucleoprotein complex subunit 4 (DKC1), fibrillarin (FBL), and nucleolar protein 58 (NOP58).^116^ These findings align with prior studies showing conserved enrichment of ribosome biogenesis proteins in N interactomes across seven coronaviruses.^52^

Building on this, several proteins identified in our study have been previously reported as targets of viral factors that localize to the nucleolus. For example, the Tat protein of human immunodeficiency virus interacts with FBL and U3 snoRNA, inhibiting pre-rRNA processing and significantly reducing cytoplasmic ribosomes.^117^ Similarly, PRRSV N has been suggested to modulate the rRNA pathway through its interaction with FBL.^40^ More broadly, RNA viruses are known to interfere with key factors in rRNA biogenesis, leading to nucleolar stress.^118^ Decreased rRNA content, which serves as a scaffold for nucleolar proteins, causes nucleolar disruption and the redistribution of these proteins to other cellular compartments. The prevailing hypothesis suggests that these changes may facilitate viral access to these proteins and their exploitation during replication.^118^ Supporting this idea, the matrix proteins of Henipaviruses and the P3 protein of Rabie virus interact with TCOF1, leading to rRNA transcriptional silencing, a process thought to mimic nucleolar stress in the host cell.^119,120^ Similarly, the NS1 protein of H3N2 IAV binds to NCL, preventing it association with the rRNA promoter, thereby inhibiting rRNA transcription and triggering nucleolar stress.^121^

Among the proteins identified in the rRNA pathway in our study, MYBBP1A exhibited a remarkably high enrichment ratio (∼400-fold), suggesting a strong interaction with PRRSV N protein. This was confirmed by Co-IP assay and found to be partially mediated by RNA. Furthermore, MYBBP1A knockdown led to a significant (>80%) reduction in viral production, indicating its role as a host dependency factor for PRRSV infection. MYBBP1A plays a dual role in the rRNA pathway, coordinating rRNA synthesis with cell growth.^110^ It interacts with Pol I, acting as a corepressor of rDNA transcription while also serving as a component within the preribosome complex to facilitate rRNA processing and ribosome assembly.^110^ Additionally, MYBBP1A has been shown to link nucleolar stress with cell cycle arrest and apoptosis through p53 activation.^122^ Given its strong association and colocalization with the N protein in the nucleolus, along with its multifaceted roles in the rRNA pathway, we hypothesize that PRRSV N-MYBBP1A interaction may represent a novel viral-host nucleolar interface, potentially regulating rRNA biogenesis. Further studies are warranted.

Beyond PRRSV, a recent computational study integrating comprehensive SARS-CoV-2 omics and CRISPR knockout screens from both SARS-CoV-2 and IAV identified MYBBP1A as a broad-spectrum, host-based synthetic lethal antiviral target.^123^ Moreover, proteomics screens have also identified MYBBP1A as an interactor of coronavirus N proteins (Table S5).^47,53^ Virus-host interactomics studies have further implicated MYBBP1A in interactions with flavivirus capsid proteins,^124,125^ the NS5B protein of HCV,^126^ and the VP35 protein of Ebola virus,^127^ underscoring its broad significant in RNA virus infections.

### 4.3 PRRSV N Interactions with RNA Methylation Enzymes

Recent research has highlighted the critical role of RNA modifications in viral infections, regulating both cellular and viral RNAs. Beyond their canonical functions, RNA methylation enzymes also modulate innate immunity, which have garnered significant attention in recent years.^128–130^ In our study, we identified an enrichment of RNA methyltransferases within the PRRSV N interactome, including rRNA 2′-O-methyltransferases such as FBL and FTSJ3. Additionally, our study confirmed interactions between PRRSV N and two NSUN family members, NSUN2 and NOP2, of which catalyze C5 methylation of RNA cytosines (m^5^C). The interaction between NSUN2 and PRRSV N was found to be strongly RNA-dependent, suggesting a possible role for viral or host RNA in facilitating this association. While the nucleolar NOP2 primarily methylates a single cytosine in 28S rRNA, NSUN2 acts on a broader range of RNA substrates in multiple cellular compartments, including the cytosol and mitochondria, where it contributes to processes such as mitochondrial tRNA methylation and the regulation of mRNA sorting into exosomes.^131–134^

A recent study reported widespread m^5^C modifications in PRRSV genomic and sg mRNAs, with enrichment near the translational start codon of sg mRNAs.^135^ Given that *Arteriviridae* viruses lack intrinsic methyltransferase activity,^136^ they likely depend on host m^5^C methyltransferases for these modifications. These observations raise the possibility that NSUN2 may catalyze the m^5^C methylation of PRRSV RNA, potentially through RNA-dependent interactions with the N protein.

NSUN2 has been widely shown to methylate viral RNA, with its effects varying by virus and methylation sites. For instance, NSUN2-mediated m^5^C modifications in hepatitis B virus (HBV) RNA contribute to viral RNA stability and replication at one conserved site,^137^ while at another, they promote nuclear export of viral mRNA, enhance translation, and help the viral RNA evade RIG-I recognition.^138^ Meanwhile, NSUN2 also facilitates type I IFN production by methylating type I IFN mRNA, which may explain its downregulation during HBV infection.^138^ In enterovirus 71, NSUN2-mediated m^5^C modifications within the IRES motif of the 5′ UTR and the coding region enhance translation and RNA stability, while mutations at these sites attenuate viral replication and pathogenicity.^139^ In contrast, NSUN2-dependent m^5^C methylation of SARS-CoV-2 genomic RNA is generally detrimental, reducing RNA stability and transcript levels, though certain methylation sites mildly enhance stability.^140^ Notably, NSUN2 knockout mice exhibited more severe pathogenesis and NSUN2 expression is down-regulated by SARS-CoV-2 infection.

In conclusion, NSUN2 plays a complex dual role in viral replication, either promoting or hindering viral RNA stability and/or translation depending on the context. Further research is needed to elucidate the specific functions of NSUN2 in the PRRSV RNA methylation and its potential impact on viral replication.

### 4.4 PRRSV N and its Possible Roles in Regulating Chromatin Structure and DDR

The identification of chromatin-associated proteins, including linker histone H1 isoforms, the FACT complex, components of DNA damage-sensing pathway such as DNA repair protein complementing XP-C cells (XPC) and poly [ADP-ribose] polymerase 1 (PARP1), as well as components of the DNA repair system, including the RFC complex and the Ku heterodimer of the DNA-PK complex, raises questions about the potential involvement of the N protein in chromatin remodeling and DDR pathway. Our study identified two isoforms of histone H1 with high enrichment ratios (>400-fold), alongside all subunits of FACT, RFC, and Ku, indicating a strong association between PRRSV N and these complexes. Notably, these DNA-binding proteins are also extensively identified in coronavirus N interactome studies (Table S5),^46,47,53,54,60,78^ however, the functional significance of these interactions in the context of infection remains unknown.

During viral infection, host chromatin structure and gene expression undergo dynamic changes to establish an antiviral response. Histone H1 and the FACT complex are key regulators of chromatin structure and transcription, including the expression of antiviral genes. Histone H1 maintains a silent state at promoter regions of IFN-stimulated genes (ISGs), but its occupancy decreases upon IFN stimulation, allowing ISG expression.^141^ The heterodimeric FACT complex, consisting of the subunits SPT16 (SUPT16H) and SSRP1, facilitates nucleosome remodeling and represses ISG expression through epigenetic mechanism.^142^ Inhibiting SUPT16H upregulates ISGs/IFNs and restricts RNA virus replication, including Zika, IAV, and SARS-CoV-2.^142^ PRRSV infection is known to disrupt IFNs/ISG expression, cytokines, and immune-related factors.^22^ By targeting chromatin-remodeling factors, the N protein may facilitate PRRSV to evade host antiviral defense.

Another important observation in our interactome is the enrichment of proteins involved in DNA damage and repair. Virus-induced genome instability is a well-documented phenomenon,^143–145^ and in response to DNA damage, the DDR pathway is activated to regulate cell cycle progression, replication fork stability, and DNA repair.^144^ While the role of DDR is well-studied in DNA viruses, its connection to RNA viruses remains less understood. However, growing evidence suggests that RNA viruses actively activate the DDR pathway to influence host cell cycle progression.^145–147^ This strategic interference can serve multiple purposes that ultimately benefit viral replication.^145–147^ Notably, DDR activation has been shown to exert a positive effect on PRRSV infection, with DDR induction occurring alongside an increase in N protein expression in infected cells.^148^ These observations provide a basis for further investigation into the potential role of PRRSV N in modulating the host DDR pathway during infection.

## 5 Concluding Remarks

The intricate network of virus-host interactions plays a crucial role throughout the viral replication cycle. Identifying host proteins that interact with viral components can provide valuable insights into the key factors driving viral replication and spread. The information gained from such studies could pave the way for the development of targeted interventions, leveraging the extensive interaction networks between viruses and their hosts. Our study focused on the N protein interactome of PRRSV, revealing its influence on key cellular processes such as ribosome function, translation, ribosome biogenesis, mRNA splicing, chromatin remodeling, and the DNA damage response pathway. Additionally, we identified PRRSV N’s interactions with regulators of the viral RNA-sensing pathway and components of cell-intrinsic antiviral mechanisms, which may contribute to its immunomodulatory effects. Notably, our findings show similarities between the PRRSV N interactome and that of other Nidoviruses, suggesting shared host factors that could be targeted in antiviral strategies. As research into PRRSV-host protein–protein interactions continue to expand, these insights may lead to the development of novel therapeutics, ultimately helping to mitigate the significant economic impact of PRRSV in the swine industry.

## 6 Limitation of the Study

Our interactome analysis was conducted in HEK293T cells, which are commonly used as a mammalian cell model for discovering the PRRSV protein interactome.^37,38,149,150^ Despite not representing a PRRSV infection model, the cells offer advantages such as efficient viral protein expression and a diverse proteome, enabling the identification of host factors and pathways potentially targeted by PRRSV during infection. Considering that the majority of our identified proteins have been found to interact with PRRSV or other Nidovirus N proteins from N-overexpressing or infected cells, we believe that our analysis can reveal potential host factors and pathways hijacked by PRRSV and provide a rich source of virus-host interactions, especially common ones across the virus order, exploitable for antiviral therapeutic interventions.

While our study provides insights into potential interactions and pathways involving PRRSV N protein, based on our analysis in a cell model, it is important to acknowledge that these findings offer a theoretical framework. The interactions identified might represent a glimpse of the possible effects of N in an infected cellular environment. However, for a comprehensive understanding of their occurrence and significance within actual viral-infected cells, further investigations conducted in the context of infection are imperative.

## Supplementary Material



## Data Availability

The mass spectrometry proteomics data have been deposited to the ProteomeXchange Consortium via the PRIDE partner repository with the data set identifier PXD0044394.

## Notes

Figure S1 and S2: Full blot corresponding to those shown in Figure 4 and Figure 6, respectively; Figure S3: Alternative models of the PRRSV N-RTCB-tRNA complexes; Table S1: Primers used for the construction of HA-tagged N interacting proteins; Table S2: Target sequences and shRNA-coding oligonucleotides (PDF); Table S3: A list of all protein groups identified at 1% FDR together with their LFQ intensities (XLSX); Table S4: A nonredundant list of identified peptides meeting the 1% FDR cutoff together with their intensities (XLSX); Table S5: A list of PRRSV N-interacting proteins identified in this study together with their enrichment ratios (XLSX); Table S6: A list of genes annotated to each GO term and the p-values associated with each annotation term (XLXS) (ZIP)
